# Decoding the past and future of distant metastasis from papillary thyroid carcinoma: a bibliometric analysis from 2004 to 2023

**DOI:** 10.3389/fonc.2024.1432879

**Published:** 2024-09-05

**Authors:** Jiaxi Wang, Mingzhu Yan, Hanqing Liu, Chuang Chen

**Affiliations:** ^1^ Department of Breast and Thyroid Surgery, Renmin Hospital of Wuhan University, Wuhan, China; ^2^ Information Center, Renmin Hospital of Wuhan University, Wuhan, China; ^3^ Department of Thyroid Surgery, The First Affiliated Hospital, School of Medicine, Zhejiang University, Hangzhou, China

**Keywords:** papillary thyroid carcinoma, distant metastasis, bibliometric, interdisciplinary, scientific structure, PTC

## Abstract

**Background:**

Papillary thyroid carcinoma (PTC) is the most common thyroid malignancy, and its distant metastasis (PTCDM), although uncommon, seriously affects the survival rate and quality of life of patients. With the rapid development of science and technology, research in the field of PTCDM has accumulated rapidly, presenting a complex knowledge structure and development trend.

**Methods:**

In this study, bibliometric analysis was used to collect 479 PTCDM-related papers published between 2004 and 2023 through the Web of Science (WoS) Core Collection (WoSCC) database. Keyword clustering analysis was performed using VOSviewer and citespace, as well as dual-map overlay analysis, to explore knowledge flows and interconnections between different disciplines.

**Results:**

The analysis indicated that China, the United States, and South Korea were the most active countries in conducting research activities. Italy’s research was notable due to its higher average citation count. Keyword analysis revealed that “cancer,” “papillary thyroid carcinoma,” and “metastasis” were the most frequently used terms in these studies. The journal co-citation analysis underscored the dominant roles of molecular biology, immunology, and clinical medicine, as well as the growing importance of computer science in research.

**Conclusion:**

This study identified the main trends and scientific structure of PTCDM research, highlighting the importance of interdisciplinary approaches and the crucial role of top academic journals in promoting high-quality research. The findings not only provide valuable information for basic and clinical research on thyroid cancer but also offer guidance for future research directions.

## Introduction

Thyroid cancer is a common type of cancer and ranks as the most prevalent endocrine malignancy worldwide (1, 2). Papillary thyroid carcinoma (PTC) accounts for the majority of all cases of thyroid malignancy (3). Although the prognosis for PTC is generally favorable, distant metastasis is a significant cause of death among patients with PTC (4). Even in papillary thyroid microcarcinoma (PTMC), distant metastasis can be fatal (5). The prognosis for patients with papillary thyroid carcinoma distant metastasis (PTCDM) typically depends on the patient’s age, the site of metastasis, and the iodine affinity of the metastasis (6).

The most common sites of distant metastasis for PTC are the lungs and bones, accounting for more than 90% of PTCDM cases (7, 8). Though rarer, PTC can also metastasize to the breast, pancreas, liver, muscles, cerebellum, uterus, and kidneys. These metastatic sites are often associated with a poor prognosis (8–15).

Researchers have consistently shown high interest in the field of PTCDM, the focus and methods of research have evolved over time with technological advancements, including risk factors, diagnostic techniques, and treatment options (16–19). However, given the vast amount of literature available, readers without appropriate reading and analysis strategies are likely to become overwhelmed by the extensive data, thereby missing key insights in the field. For researchers new to this area, quickly grasping representative scholars, articles, research hotspots, and developments is particularly challenging.

Bibliometrics, first proposed by Alan Pritchard in 1969, is defined as ‘the application of mathematical and statistical methods to books and other media of communication to reveal the process of information handling and the nature and trends of development within a discipline’ (20). Bibliometrics offers a quantitative approach to review and investigate the existing literature in a field (21). By applying bibliometric and its visualization techniques, we can swiftly identify literature development trends, high-quality authors, and institutions in the PTCDM field, thereby determining credible research topics and accelerating the research process (22).

Although PTCDM has garnered considerable attention, the bulk of the existing literature remains concentrated on case reports and metastases to specific sites. These studies did not analyze and summarize the developmental trends, principal authors, and research hotspots of PTCDM (23–27). To date, no scholars have employed bibliometric methods to systematically analyze the literature on PTCDM. Over the past 20 years, the PTCDM field has transformed significantly. Early 21st-century advancements in PET technology improved detection of cancerous and metastatic lesions, sparking interest in distant metastasis studies, including PTCDM (28–33). Concurrently, high-throughput sequencing advancements, particularly RNA-seq and proteomics, provided tools to explore PTCDM mechanisms deeply (19, 34, 35). These technological advances led to a surge in research, including clinical case reports and molecular mechanism studies. Publications before 2004 are sparse and lower in quality, while literature from 2024 is still emerging and not yet comprehensive. This study aims to analyze PTCDM literature from 2004 to 2023 using bibliometric methods and employ visualization tools to display these analyses, thereby elucidating the historical and future trends of PTCDM. We will examine the trends in annual publications, identify the most prolific and most cited journals, authors, and countries, and highlight the most frequently used keywords and most relevant articles. This will reveal the developmental dynamics of the field, explore research priorities, and provide guidance for future research directions.

## Materials and methods

### Source of data

**Figure 1:**
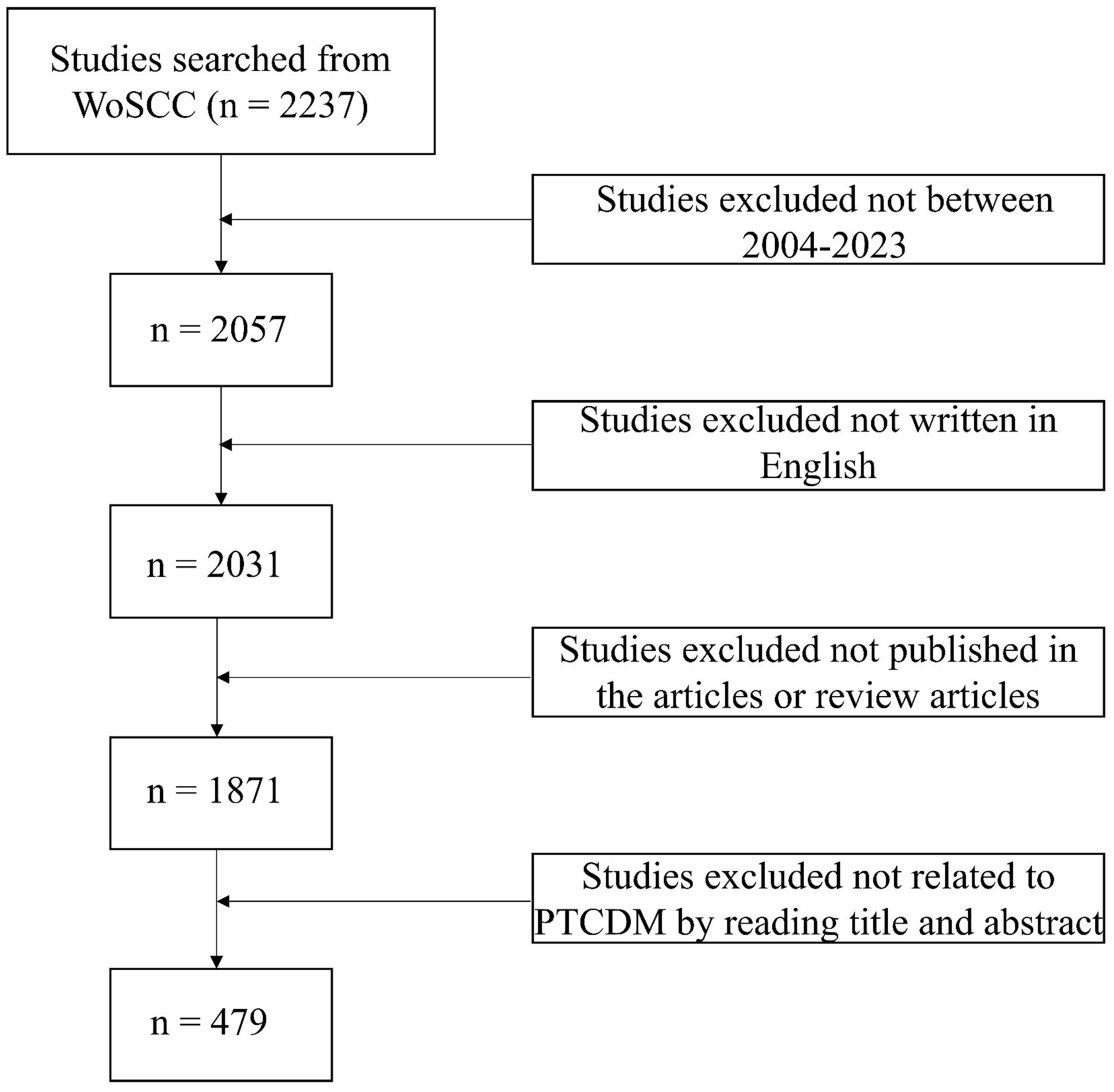
Detailed flowchart for searching and filtering.

This study utilizes the Web of Science (WoS) Core Collection (WoSCC) database as its data source. Recognized as a high-quality literature resource, WoS is accepted by many researchers and includes a wide range of publications across various fields, making it highly suitable for bibliometric analysis (21, 36, 37). To ensure the comprehensiveness and accuracy of the search results, the Science Citation Index Expanded (SCI-EXPANDED) was selected as the index. Data was extracted from WoSCC on February 3, 2024. The final search strategy defined for this study was TS = ((“Papillary Thyroid Carcinoma” OR “Papillary Thyroid Cancer”) AND (“Metastasis” NOT (“Lymphatic Metastasis” OR “Lymph Node Metastasis”))), covering the period from January 2004 to December 2023, and limiting document types to Articles and Review Articles. After removing duplicates, a total of 479 journal papers were obtained, including 430 Articles and 49 Review Articles. To enhance transparency and reproducibility, inclusion criteria were set to articles and review papers published between January 2004 and December 2023, indexed in SCI-EXPANDED, and containing specified search terms in the title, abstract, or keywords. Articles focusing solely on lymph node metastasis or those not specifically on PTCDM were excluded to narrow the focus to distant metastasis. The dataset was downloaded in “plain text file” format and then analyzed using Microsoft Excel (version 2108; Microsoft Corporation, Redmond, WA, USA) and VOSviewer V1.6.20 (Center for Science and Technology Studies, Netherlands) software, along with CiteSpace V6.3.1 (**Figure 1**).

### Statistical analysis

The development of a field can be understood through bibliometric analysis, which provides detailed insights into authors, keywords, journals, countries, institutions, and references (38). This study applies Lotka’s Law and Price’s Law to analyze the distribution of annual publications from 2004 to 2023 and to identify key authors (39, 40). Each year’s publication count is visualized, and the fit to an exponential growth phase is assessed using the coefficient of determination (R^2^). Bradford’s Law is utilized to determine the core journals within the field, which are significant due to their high volume of publications, reflecting their centrality and importance. The Journal Citation Reports (JCR) categories, provided by Clarivate Analytics based on citation data in the WoS database, is a system that assesses the relative importance of scientific and social sciences journals. Journals are evaluated and categorized mainly through the Impact Factor (IF), which ranks them within their disciplinary fields from Q1 to Q4, indicating their relative standing and influence (41). To emphasize the importance of the journals in their respective fields, this study incorporates JCR categories into the journal analysis.

Visualization methods in bibliometrics facilitate data interpretation, making the results more intuitive and comprehensive. This study uses VOSviewer and CiteSpace for creating knowledge maps. VOSviewer, a widely-used scientific visualization software based on probabilistic data normalization techniques, is designed for creating, browsing, and analyzing networks of scientific publications, keywords, co-authorships, and more. By utilizing VOSviewer, we can visually display key research themes and their interconnections within a scientific field, identify hotspots and trends, and recognize core journals, leading researchers, and primary institutions. Its visualization capabilities provide researchers with powerful tools for understanding the structure and evolution of scientific knowledge (42). Each node on the VOSviewer map corresponds to a specific parameter, such as a country/region, journal, paper, or keyword, with the size determined by metrics like publication count, frequency of occurrence, or citation count. Clusters color the nodes, and the Total Link Strength (TLS) index assesses the strength of connections. CiteSpace, on the other hand, employs a set theory-based data normalization approach for measuring the similarity of knowledge units. Its similarity algorithm is used to produce timelines within time slices, clearly outlining the evolutionary process of knowledge and the historical span of the literature within a cluster. Moreover, keyword citation bursts can reveal recurrently used keywords and their emergence times (43). Additionally, this study uses Tableau (version 2024.1) software to visually represent the geographic distribution of published articles, with map information sourced from the software itself.

## Results

### Annual publications grow exponentially

**Figure 2:**
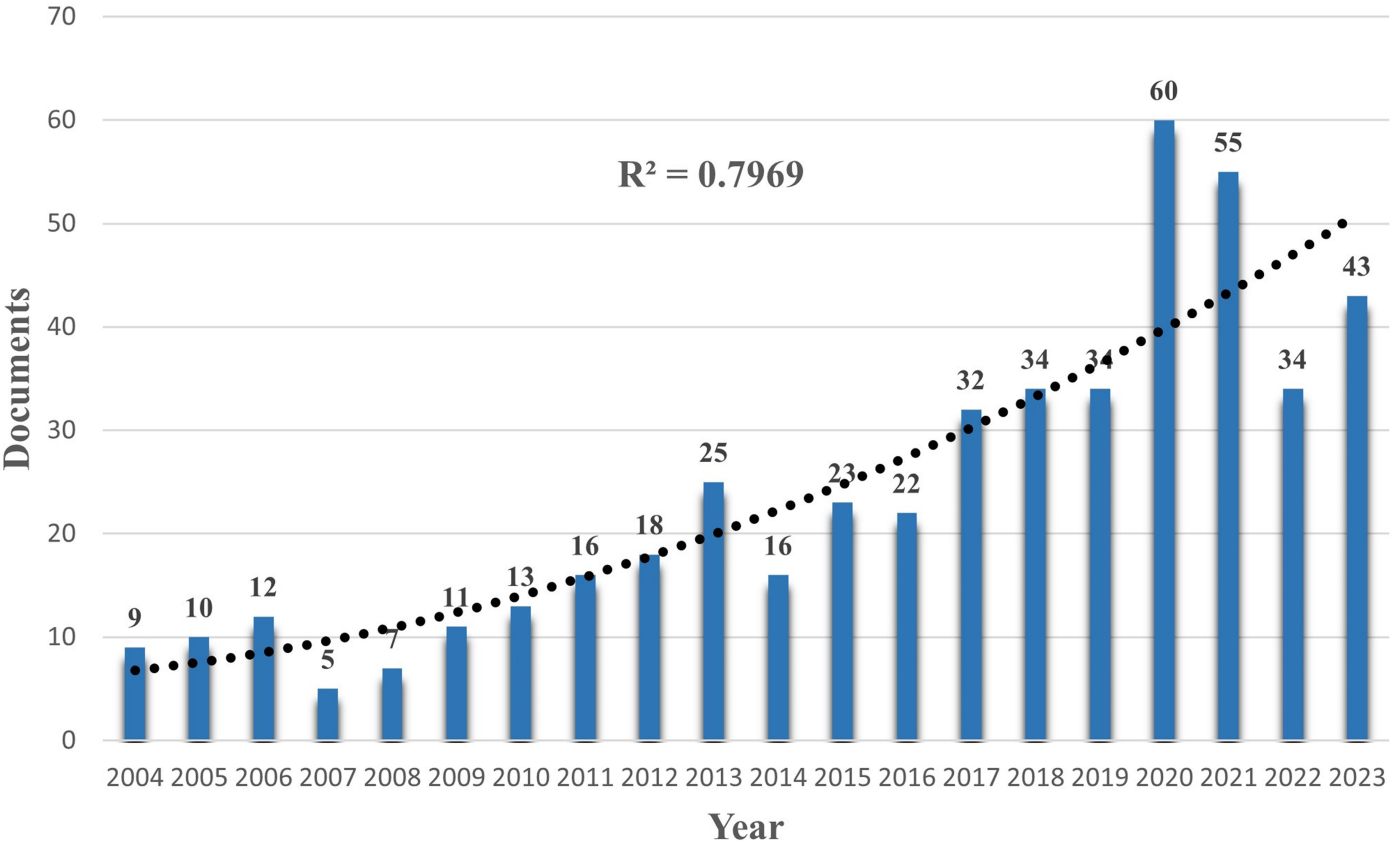
Number of annual research papers and growth trends of PTCDM from 2004 to 2023.

This study compiled 479 documents covering research published from January 2004 to December 2023, including 430 research papers and 49 review articles. These publications were authored by 2,905 researchers from 623 institutions across 43 countries and were published in 242 different journals. They referenced 11,810 articles from 2,029 journals, contributed by 8,999 researchers. An examination of the annual publication volume shows that the number of publications in the PTCDM field has been rising exponentially, particularly after 2016, with a sharp increase noted. In 2020 and 2021, the publication volume stabilized at over 50 articles each year, indicating increasing scholarly attention in recent years and establishing it as a new focal point within the PTC research field. In 2022, there was a sudden decrease in publication volume compared to 2021, followed by an increase in 2023. This fluctuation may suggest a waning interest in PTCDM research, possibly influenced by the COVID-19 pandemic, which led to a reallocation of scientific resources and focus. Nevertheless, this area remains a significant and attention-worthy research direction within the PTC-related fields (**Figure 2**).

### Core author

According to Lotka’s law, there is a relationship between the number of scientific workers and the number of papers they produce. Price’s law asserts that the most active scientists in a field, who typically contribute over half of the scientific output, number approximately the square root of the total number of scientists in that field. After analyzing and computing, we identified core authors as those who have published two or more papers, totaling 258 core authors. **Table 1** displays the authors who have published more than four papers in the field, categorized as highly productive authors. The three authors with the highest number of publications (Documents = 7) are Luo, Quan-Yong; Miyauchi, Akira; and Qiu, Zhong-Ling. The author with the highest number of citations (254 citations) is Miyauchi, Akira, while the highest average citations per paper (average of 41.2 citations per article) is held by Ito, Yasuhiro.

**Table 1:** Highly productive authors with more than 4 publications in the field of PTCDM.

Rank	Author	Documents	Citations	Average Citation	H-index
1	luo,quan-yong	7	123	17.6	27
2	miyauchi,akira	7	254	36.3	71
3	qiu,zhong-ling	7	123	17.6	20
4	ge,minghua	6	41	6.8	26
5	ito,yasuhiro	6	247	41.2	52
6	lin,jen-der	6	156	26.0	34
7	song,hong-jun	6	103	17.2	96
8	chao,tzu-chieh	5	150	30.0	20
9	jeon,min ji	5	67	13.4	1
10	kim,won bae	5	67	13.4	57
11	park,cheong soo	5	66	13.2	41
12	shen,chen-tian	5	103	20.6	20
13	shong,young kee	5	67	13.4	51
14	zhang,hao	5	155	31.0	26
15	zhang,wei	5	53	10.6	7

**Figure 3:**
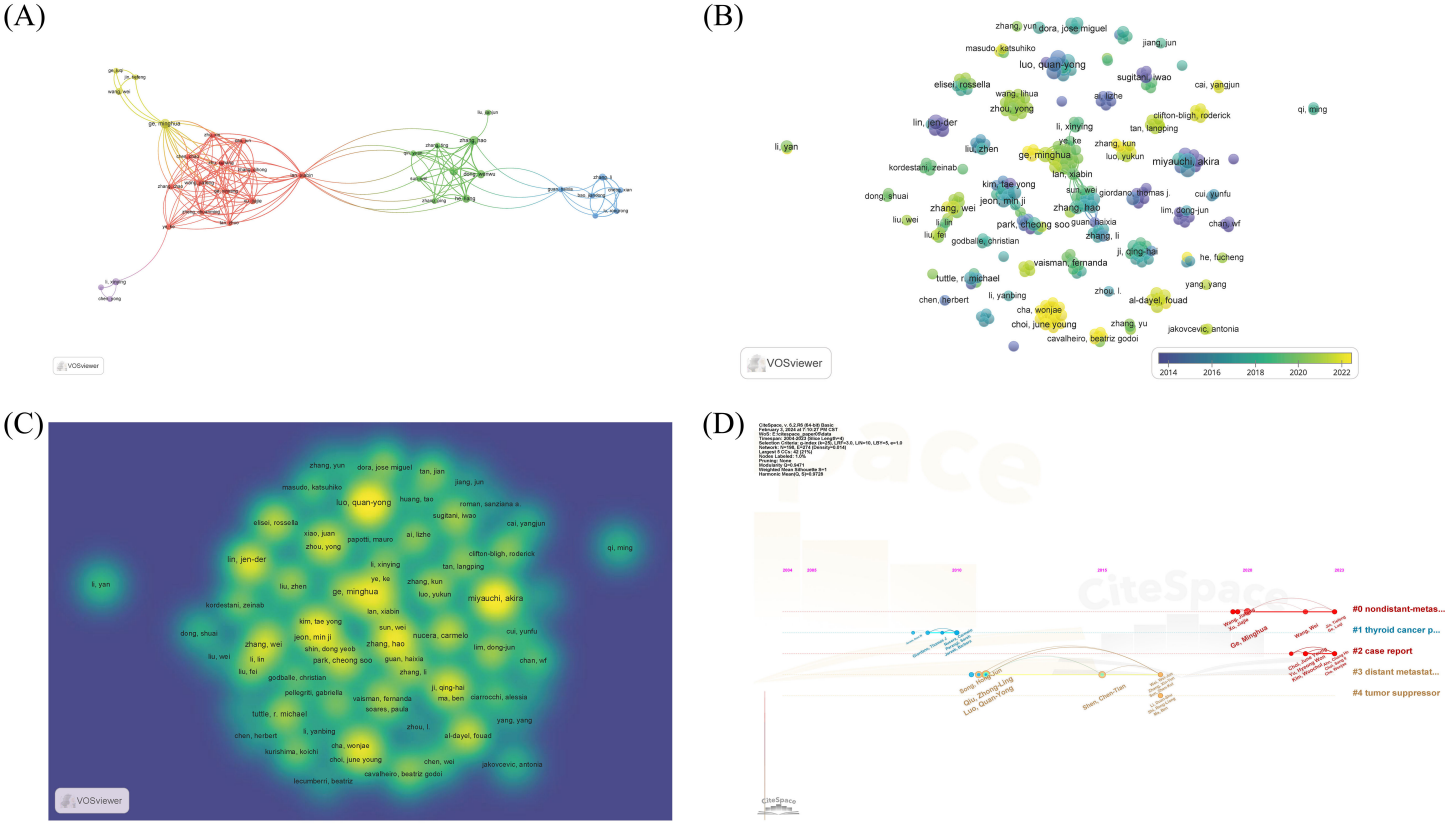
Description of authors who published papers in the field of PTCDM. **(A)** Cluster diagram of the 35 authors who collaborated most closely among the core authors. **(B)** Diagram of 258 core authors publishing papers in different years. Blue represents earlier publication time, yellow represents nearer publication time. **(C)** Density chart of papers published by 258 core authors. The larger the yellow dot, the higher the opacity, which means the author has published more papers. **(D)** Timeline chart of all authors who published papers. The size of the dot represents the amount of posts, and the position of the dot represents the time of posting.

Among the core authors, only a subset exhibits significant collaborative relationships. **Figure 3A** displays a clustering diagram of the 35 authors who collaborate most closely, with Lan, Xiabin linking the red and green clusters. The yellow cluster, led by Ge, Minghua, has published the most articles among these 35 authors. **Figure 3B** depicts the variation in publications by different authors over the years, where blue-purple represents earlier years and yellow indicates more recent publications, emphasizing the ongoing focus and research by Park Cheong Soo in the field. **Figure 3C** shows a density map of the authors, where higher opacity in yellow indicates a greater volume of publications by that author. Early research in the PTCDM field was initiated by Song Hong-Jun, while Shen Chen-Tian’s research became prominent in 2015, followed by continued in-depth studies in this area by Wei Wei-Jun and others (**Figure 3D**).

### Institution

**Figure 4:**
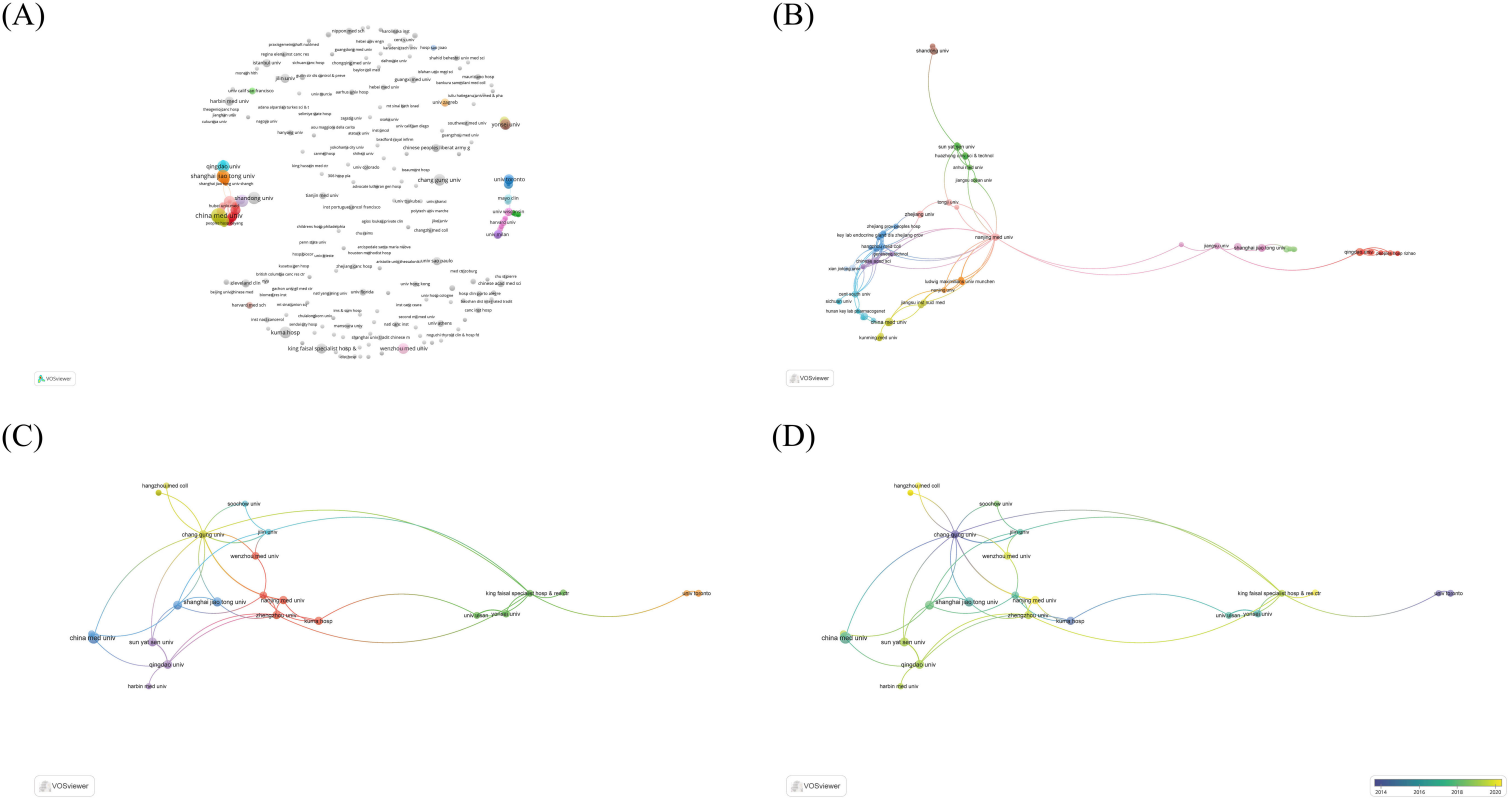
Description of institutions that published papers in the field of PTCDM. **(A)** A network of cooperation among 623 institutions. **(B)** A network of 87 institutions with the closest cooperation. **(C)** Citation network of 23 institutions with more than 5 publications. The lines between points represent mutual citations. **(D)** The publication year network of 23 institutions with more than 5 publications, blue means the publication time is early, and yellow means the publication time is recent.

This study analyzed the collaborative publishing and citation relationships among 623 institutions, dividing these relationships into 12 clusters, with the majority located in China (**Figures 4A, B**). Special attention was given to institutions that have published five or more papers. There are 23 such institutions, and the lines between points in the graph represent inter-citation relationships (**Figures 4C, D**). Among these institutions, China Medical University has the highest number of publications, with a total of 14 papers and 393 citations, averaging 28.1 citations per paper. The institution with the highest number of citations is the Karolinska Institute, with a total of 718 citations. Karolinska University Hospital has the highest average number of citations per paper; despite only having two papers, each one is cited on average 359 times (**Table 2**).

**Table 2:** The top ten institutions with the largest number of publications in the field of PTCDM.

Rank	Organization	Documents	Citations	Average Citation
1	China Medical University	14	393	28.1
2	Shanghai Jiao Tong University	10	163	16.3
3	Sun Yat-sen University	9	162	18
4	Shandong University	9	95	10.6
5	Qingdao University	9	84	9.3
6	Zhengzhou University	8	118	14.8
7	Nanjing Medical University	8	122	15.3
8	Kuma Hospital	8	264	33.0
9	Chang Gung University	8	340	42.1
10	Yonsei University	7	107	15.3

#### Country/region

**Figure 5:**
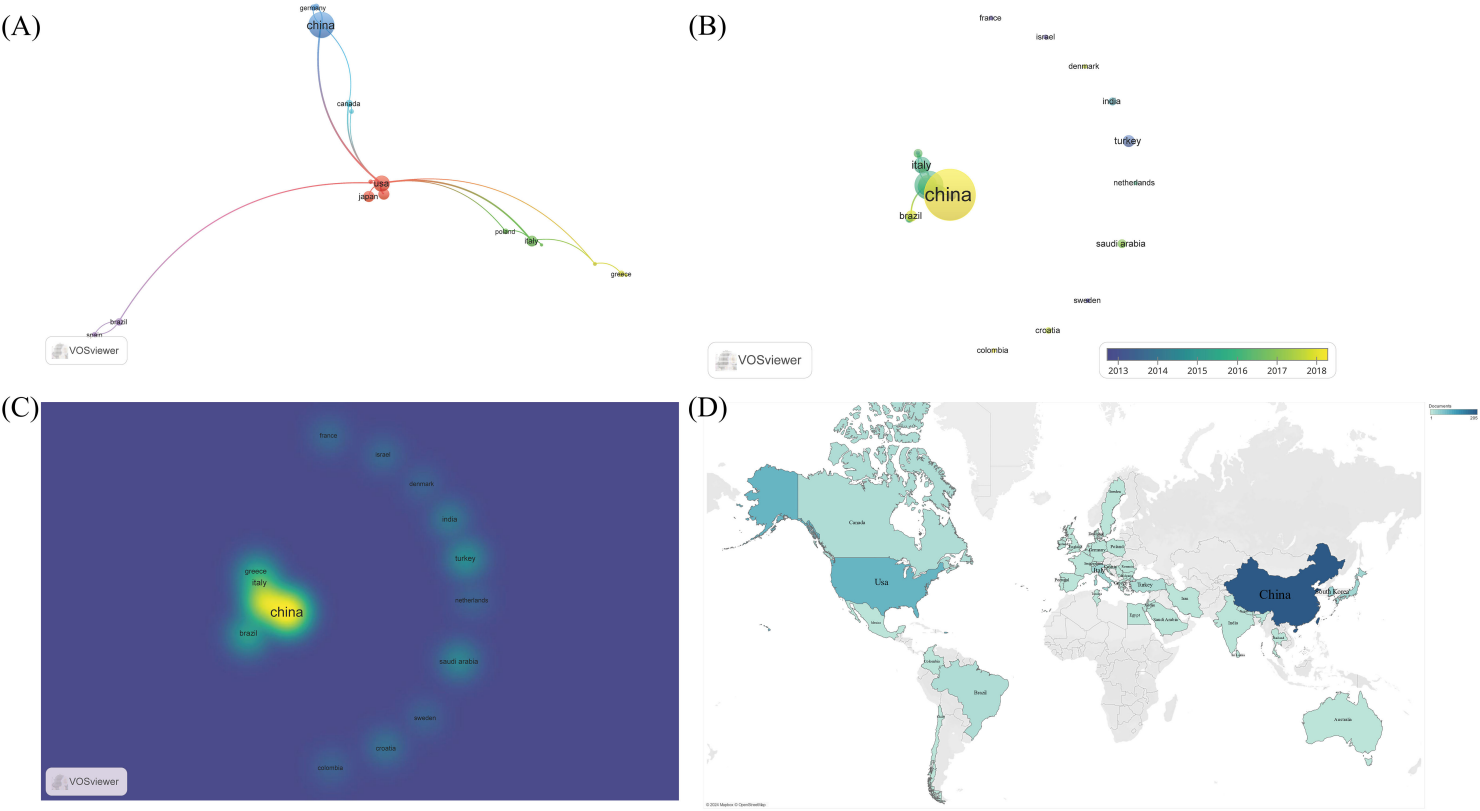
The publication status of 43 different countries/regions in the field of PTCDM. **(A)** Cluster network of 18 closely cooperating countries/regions. The color represents the cluster, the size of the dot represents the volume of publications, and the connection represents the cooperative relationship. **(B)** Cluster network of 43 countries. **(C)** Density chart of the number of publications in 43 countries. **(D)** The publication volume of 43 countries is displayed on a world map. The darker the blue, the greater the volume of posts.

In the research field of PTCDM, the involved authors hail from 43 different countries and regions. According to our data, China leads with 205 papers, followed by the USA with 73 documents. In terms of citation numbers, China is ahead with 3301 citations, while Italy boasts the highest average citation rate, with each paper being cited 31.1 times on average (**Table 3**). There are 29 countries/regions that have published two or more papers, 18 of which show significant tendencies towards international collaboration. Researchers from the USA have the closest collaborative ties, working with scholars from 9 different countries/regions, followed by Italy, which has collaboration with 4 countries/regions. Among these 29 countries/regions, Turkey and Germany were among the early ones to focus on the PTCDM field, but in recent years, China and Brazil have shown increased attention to this area (**Figures 5A–C**). In **Figure 5D**, different shades of blue on the map indicate the publication volume of each country. The darker the color, the higher the publication volume, making it evident that China and the USA have published the majority of the research articles in this field.

**Table 3:** The top 5 countries/regions with the largest number of publications in the field of PTCDM.

Rank	Country	Documents	Citations	Average Citation
1	China	205	3301	16.1
2	USA	73	1831	25.1
3	South Korea	32	411	12.8
4	Japan	30	535	17.8
5	Italy	28	872	31.1

#### Key words

**Figure 6:**
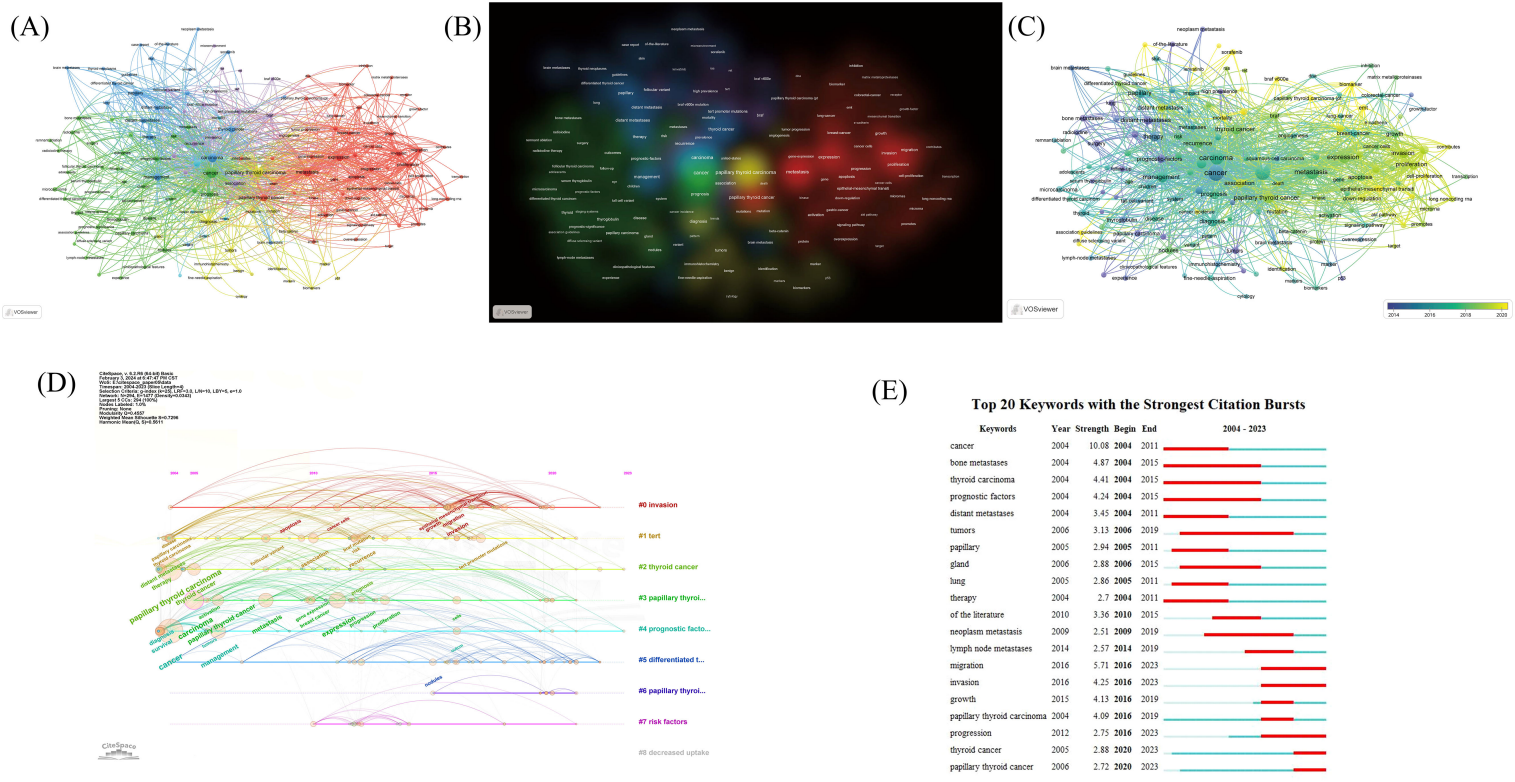
Keywords network and burst time. **(A)** Clustering network of 158 commonly used keywords. **(B)** Density chart of 158 commonly used keywords. **(C)** Network of 158 commonly used keywords’ appearance time. **(D)** Timeline diagram of 1861 keywords (all keywords). **(E)** Top 20 Keywords with the strongest citation bursts.

Keywords serve to condense and summarize the themes of research. Applying Zipf’s Law, we identified 158 terms that appeared more than five times. Zipf’s Law posits that in a corpus of natural language, the frequency of a word is inversely proportional to its ranking in the frequency table. We conducted a cluster analysis of these terms using the bibliometric tool VOSviewer, resulting in six different clusters, each represented by a distinct color. Common search terms include “papillary thyroid carcinoma,” “metastasis,” “recurrence,” and “cancer.” Additionally, significant research areas related to PTCDM such as “survival,” “therapy,” “diagnosis,” “braf,” and “epithelial-mesenchymal transition” were also identified. Notably, “distant metastasis” often appears alongside terms like “braf,” “case report,” “tert promoter mutations,” “recurrence,” and “survival” (**Figure 6A**). The five most frequent keywords are “cancer,” “papillary thyroid carcinoma,” “carcinoma,” “metastasis,” and “expression” (**Figure 6B**). Keywords such as “therapy,” “thyroglobulin,” “surgery,” “radioiodine,” “diagnosis,” and “survival” all appeared before or by the year 2014. After 2010, researchers began to focus on “risk factors,” while terms like “biomarker,” “braf v600e,” “e-cadherin,” and “akt pathway” emerged around the year 2020 (**Figures 6C–E**).

#### Journal analysis

**Figure 7:**
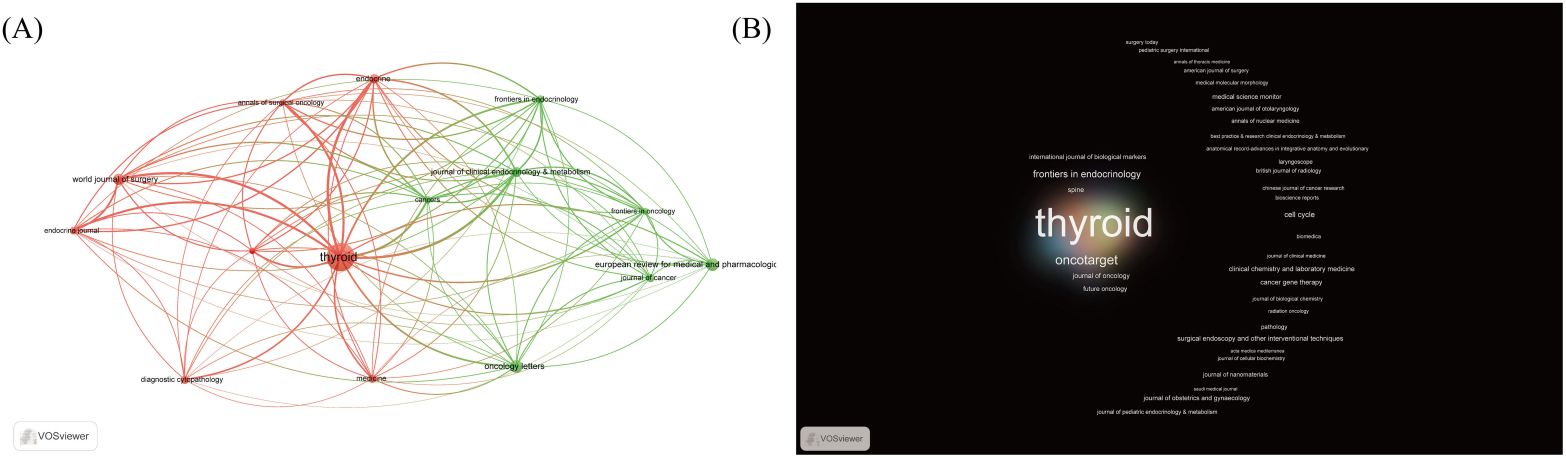
Description of journals publishing literature in the field of PTCDM. **(A)** Clustering network of 15 core journals. **(B)** Density chart of articles published in all journals, with colors representing clusters and label sizes representing the number of articles published.

According to Bradford’s Law, if scientific journals are arranged in decreasing order of the number of papers they publish in a specific discipline, they can be divided into three zones—core, secondary, and peripheral—with approximately equal numbers of papers in each zone. The relationship between the number of journals in the core zone and the subsequent zones follows a 1: a: a^2^ ratio. Our calculations show that there are 15 journals in the core zone, with an ‘a’ value of approximately 3.3 (**Table 4**). An analysis of publishing sources revealed a total of 242 journals. The journal “Thyroid,” published by Mary Ann Liebert, Inc., leads with 33 papers, accounting for 6.9% of the total publications. This is followed by “European Review for Medical and Pharmacological Sciences,” published by Verduci Publisher, and “Oncology Letters” by Spandidos Publications Ltd, each with 12 papers representing 2.5% of total publications (**Figures 6A, B**). Based on Bradford’s Law, there should be 15 core journals in the PTCDM field, accounting for 29.0% of total publications, with 33.3% of journals ranked in the first quartile of the WoS categories. Additionally, 40% of the journals have over 90% of their papers available as open access. The journals in two clusters are differentiated by focus: the red cluster mainly includes clinical journals focused on the diagnosis and treatment of PTCDM, while the green cluster consists predominantly of journals related to oncology (**Figure 7A**).

**Table 4:** 15 core journals in the field of PTCDM determined according to Bradford Law.

Journals	Documents	Citations	Average Citation	JCR	% OA
Thyroid	33	964	29.2	Q1	17.81
European Review for Medical and Pharmacological Sciences	12	164	13.7	Q2	0.016
Oncology Letters	12	140	11.7	Q3	99.58
World Journal of Surgery	10	531	53.1	Q2	15.87
Journal of Clinical Endocrinology & Metabolism	9	167	18.6	Q1	58.23
Endocrine	8	94	11.8	Q3	22.23
Diagnostic Cytopathology	7	62	8.9	Q4	8.42
Frontiers in Endocrinology	7	100	14.3	Q1	99.76
Journal of Cancer	7	83	11.9	Q2	99.50
Medicine	7	82	11.7	Q3	96.02
Cancers	6	52	8.7	Q2	99.8
Endocrine Journal	6	117	19.5	Q4	65.8
Annals of Surgical Oncology	5	246	49.2	Q1	16.6
Frontiers in Oncology	5	16	3.2	Q2	99.8
Head and Neck-journal for the Sciences and Specialties of the Head and Neck	5	59	11.8	Q1	18.2

The journal “Thyroid” is the most cited, with 964 citations, followed by “Cancer” with 540 citations, “World Journal of Surgery” with 531 citations, “Oncogene” with 270 citations, and “Endocrine-related Cancer” with 262 citations. “Cancer” also has the highest average citations per article at 540, followed by “Proceedings of the National Academy of Sciences of the United States of America” with 134, “Human Pathology” with 96, “Oncogene” with 90, and “Endocrine-related Cancer” with 87.3. The citation details for these 242 journals are displayed in a density map, where opacity correlates positively with citation counts, and colors indicate different clusters (**Figure 7B**).

#### Co-citation analysis

##### Analysis of co-cited journals

**Figure 8:**
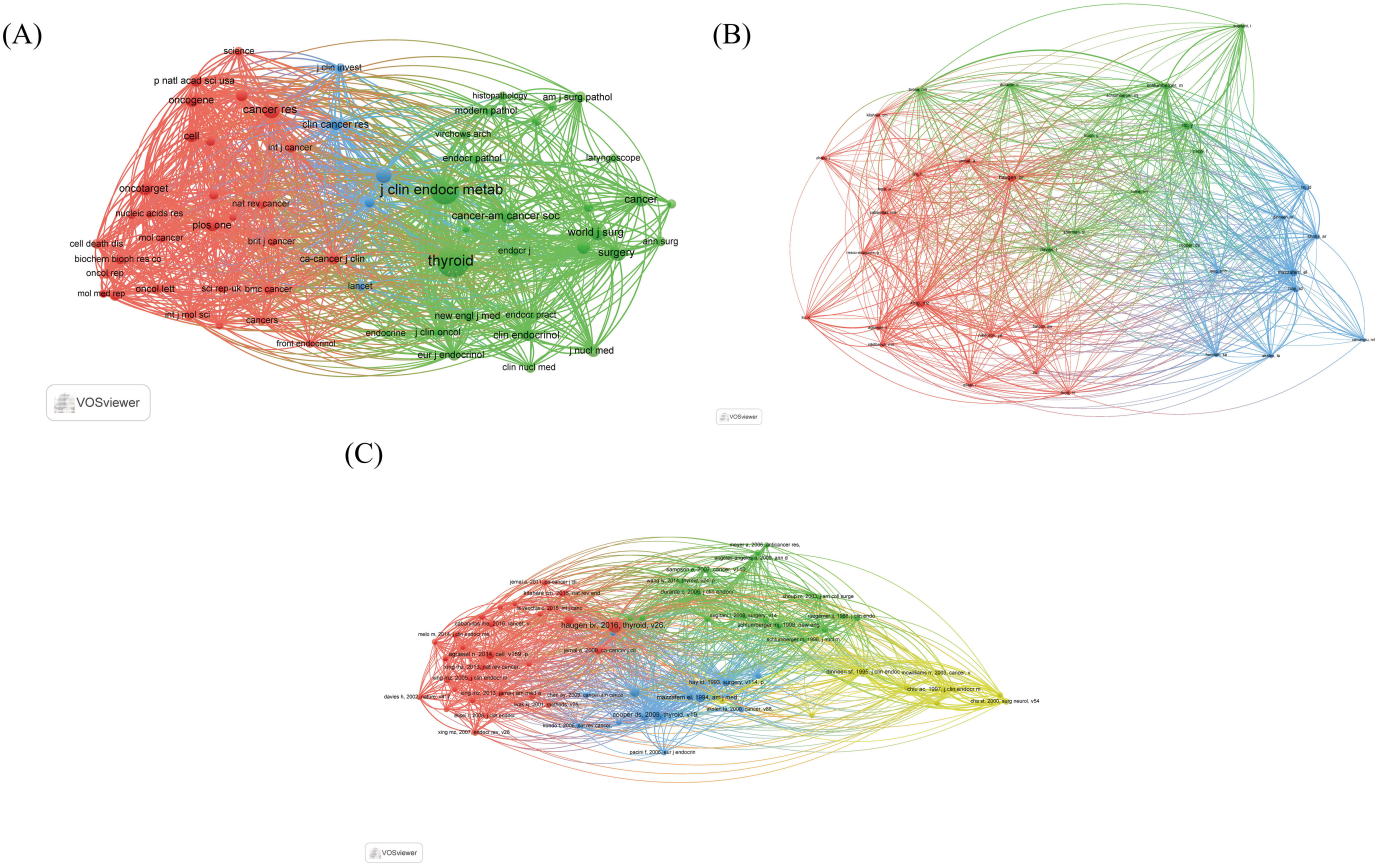
Network of citations. **(A)** Clustering network of cited journals. **(B)** Clustering network of authors of cited publications. **(C)** Cluster network of cited publications.

The 479 papers collectively cite 11,810 articles from 2,029 journals. Sixty-three journals with 50 or more citations have been identified and categorized into three clusters. The red and blue clusters are primarily associated with clinical and oncological research, providing fundamental research backgrounds and ideas for PTCDM. The green cluster mainly focuses on thyroid and endocrinology, with journals that tend to publish on the pathology and molecular mechanisms of thyroid cancer (**Figure 8A**). Among these journals, “Thyroid” has the highest citation count, with 33 articles cited a total of 964 times. “Cancer” follows, with one article cited 540 times, making it the journal with the highest average citations per article (**Table 5**).

**Table 5:** The top ten most cited journals in the field of PTCDM.

Rank	Journals	Documents	Citations	Average Citation
1	Thyroid	33	964	29.2
2	Cancer	1	540	540.0
3	World Journal of Surgery	10	531	53.1
4	Oncogene	3	270	90.0
5	Endocrine-related Cancer	3	262	87.3
6	Annals of Surgical Oncology	5	246	49.2
7	Biochemical and Biophysical Research Communications	4	211	52.8
8	Oncotarget	5	189	37.8
9	Journal of Clinical Endocrinology & Metabolism	9	167	18.6
10	European Review for Medical and Pharmacological Sciences	12	164	13.7

##### Analysis of co-cited authors

The 11,810 cited articles were authored by 8,999 researchers. An analysis revealed that 38 authors have been cited 20 times or more. These authors are divided into three clusters. Representing the red cluster is Chinese scholar Mingzhao Xing from the Southern University of Science and Technology, whose papers have been cited 116 times. The green cluster’s representative author is Japanese scholar Yasuhiro Ito from Kuma Hospital, with citations totaling 104 times. The blue cluster’s representative is American scholar E L Mazzaferri from the Division of Endocrinology at Shands Hospital, whose papers have been cited 89 times (**Figure 8B**).

##### Analysis of co-cited publications

Further analysis of the co-citations in the literature was conducted using VOSviewer, identifying the top ten cited documents in the PTCDM field from 2004 to 2023 (**Table 6**). Most highly cited documents primarily originate from the years 1995 to 2010, with studies having large sample sizes often becoming highly cited. A co-citation map was created to display the papers with citations equal to or greater than 10, totaling 71 papers. The two most cited documents both originated from the journal “Thyroid” (**Figure 8C**).

**Table 6:** Top 10 most cited publications in the field of PTCDM.

Title	Year	Category	Journal	Citations
2015 American Thyroid Association Management Guidelines for Adult Patients with Thyroid Nodules and Differentiated Thyroid Cancer: The American Thyroid Association Guidelines Task Force on Thyroid Nodules and Differentiated Thyroid Cancer	2016	Review	Thyroid	86
Revised American Thyroid Association management guidelines for patients with thyroid nodules and differentiated thyroid cancer	2009	Review	Thyroid	40
Integrated genomic characterization of papillary thyroid carcinoma	2014	Article	Cell	33
Trends in Thyroid Cancer Incidence and Mortality in the United States, 1974-2013	2017	Article	JAMA-Journal of The American Medical Association	33
Long-term impact of initial surgical and medical therapy on papillary and follicular thyroid cancer	1994	Article	AMERICAN JOURNAL OF MEDICINE	33
Increasing incidence of thyroid cancer in the United States, 1973-2002	2006	Article	JAMA-Journal of The American Medical Association	32
Distant metastases in papillary thyroid carcinoma: 100 Cases observed at one institution during 5 decades	1995	Article	Journal of Clinical Endocrinology & Metabolism	25
Papillary and follicular thyroid carcinoma	1998	Review	The New England Journal of Medicine	25
A National Cancer Data Base report on 53,856 cases of thyroid carcinoma treated in the U.S., 1985-1995	1998	Article	Cancer	24
Long-term outcome of 444 patients with distant metastases from papillary and follicular thyroid carcinoma: benefits and limits of radioiodine therapy	2006	Article	Journal of Clinical Endocrinology & Metabolism	23

#### Subject analysis

**Figure 9:**
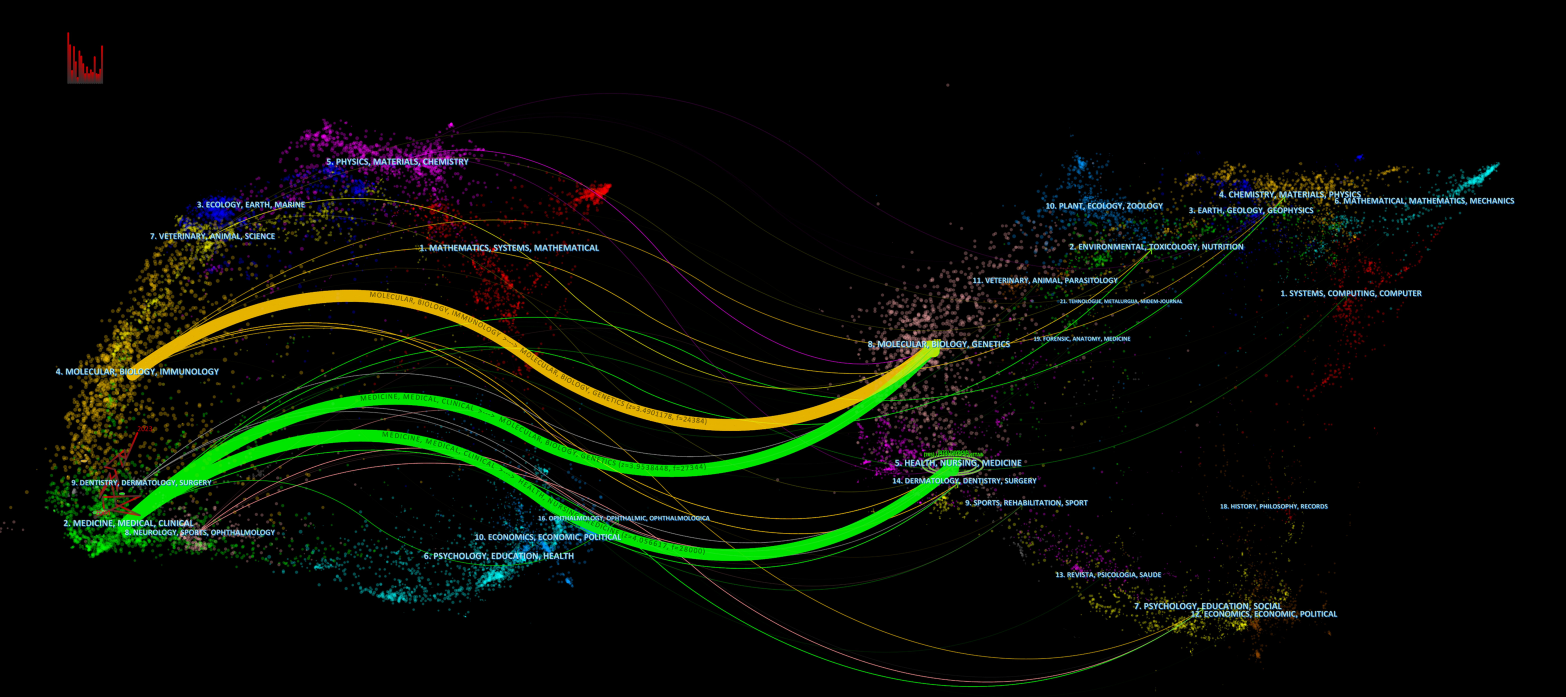
Dual-map overlay of PTCDM. The left side represents the field of research included in the article, and the right side represents the field of the article’s references.

Through dual-map overlay analysis of citing and cited journals, this study reveals a journal discipline co-occurrence map. In this map, citing journals refer to those that publish articles citing other research, while cited journals are those referenced by articles in the citing journals. The curves in the map, moving from the citing side to the cited side, demonstrate the flow of knowledge from published research to the disciplines citing this research, thereby revealing the connections and influence pathways between different research fields. The citing side predominantly clusters in the fields of molecular biology, immunology, and clinical medicine, forming major clusters like “molecular, biology, immunology” and “medicine, medical, clinical.” Correspondingly, the cited side primarily involves molecular biology, genetics, and the fields of health, nursing, and medicine, with main clusters including “molecular, biology, genetics” and “health, nursing, medicine.” Moreover, the interdisciplinary integration and crossover are particularly evident in this research field, especially in the cited journals, where categories such as “systems, computing, computer” are frequently referenced, indicating the increasingly widespread application of computer science technology in biomedical research. This interdisciplinary knowledge flow and integration deepen our understanding of the interactions between these fields (**Figure 9**).

## Discussion

In this era of rapid accumulation of scientific research results, new achievements continually emerge across various research fields. The explosive growth of scientific information makes it increasingly challenging to stay updated and master the latest research findings. To demonstrate the current global scientific accomplishments related to PTCDM, this study utilized 479 documents published between 2004 and 2023 from the WoS database, including 430 research papers and 49 review articles. Through bibliometric analysis, we have revealed and elaborated the knowledge structure of this field, providing new perspectives for understanding its scientific development.

During the time frame covered by this study, the most prolific countries/regions were China, the USA, and South Korea, while Italy had the highest average citation rate per research paper. Notably, Kakudo, K, who began researching this field in 2004, was the first scholar to delve into this area. From 2004 to 2006, the number of publications on this topic increased annually, despite a decline in 2007, followed by continuous growth. Particularly in 2020 and 2021, there was a significant surge in research in this field. This increase may be linked to the dramatic rise in hospital check-ups during the COVID-19 pandemic, which led to the detection of more late-stage PTC patients. Researchers have focused on this trend and conducted in-depth studies on PTCDM. Additionally, studies suggest that the increased aggressiveness of PTC may be associated with the COVID-19 pandemic (44). It can be seen that the COVID-19 pandemic has a huge impact on the healthcare sector (45, 46).

This study analyzed 2,905 authors, among which 258 were identified as core authors based on their publication volume. Luo Quan-yong from Shanghai Jiao Tong University Affiliated People’s Hospital published the most, with an H-index of 27. His research into PTCDM began in 2011, with his earliest publication titled “*Brain metastases with exceptional features from papillary thyroid carcinoma: report of three cases.*” His most recent publication, appearing in 2020 in “Frontiers in Endocrinology,” is titled “*Lung Metastases From Papillary Thyroid Cancer With Persistently Negative Thyroglobulin and Elevated Thyroglobulin Antibody Levels During Radioactive Iodine Treatment and Follow-Up: Long-Term Outcomes and Prognostic Indicators.*” This article suggests that only the affinity for I-131 is correlated with a 10-year progression-free survival (PFS) rate in patients with lung metastases from PTC (47). Luo’s work spans case reports and retrospective clinical studies, revealing some clinical features of PTCDM patients but lacking an exploration of the molecular mechanisms behind these characteristics. Similarly, Miyauchi Akira from Kuma Hospital, Dept of Surgery, Kobe, Hyogo, Japan, who has also published seven papers and has the highest number of citations and average citations per paper among these authors, holds an H-index of 71. His earliest work on PTCDM, published in the “Journal of Clinical Pathology,” is titled “Parathyroid invasion, nodal recurrence, and lung metastasis by papillary carcinoma of the thyroid.” This paper highlights that parathyroid invasion may be an important histological feature of PTC, indicating a greater likelihood of lymph node recurrence and lung metastasis (48). His most cited work, “Overall Survival of Papillary Thyroid Carcinoma Patients: A Single-Institution Long-Term Follow-Up of 5897 Patients,” published in 2018 in the “World Journal of Surgery”, has been cited 147 times to date (49). His highest impact factor publication in the PTCDM field is *“Control of Lung Metastases and Colon Polyposis with Lenvatinib Therapy in a Patient with Cribriform-Morular Variant of Papillary Thyroid Carcinoma and an APC Gene Mutation: A Case Study”*, published in “Thyroid.” This article reveals the promising potential of Lenvatinib in treating lung metastases from PTC and familial adenomatous polyposis (50). Despite considerable research by these authors into PTCDM, most studies remain focused on the disease characteristics or patient treatment aspects, with few groundbreaking results on the factors that promote or inhibit the metastasis of PTC.

This study analyzed literature from 43 countries/regions, with the highest number of documents originating from China. However, Italy stood out in terms of average citations per paper. Notably, the research by Elisei, Rossella titled *“Correlation between B-RAFV600E mutation and clinico-pathologic parameters in papillary thyroid carcinoma:: data from a multicentric Italian study and review of the literature,”* was cited 197 times in the WoS-indexed literature and 210 times across all databases. This paper discusses the association of the B-RAFV600E mutation with tumor growth patterns but found no link to poorer outcomes, including distant metastases (51). Interestingly, more research supports that the B-RAFV600E mutation is closely related to distant metastases and poor prognosis in thyroid cancer, and some studies even suggest that this mutation can independently predict the recurrence risk for patients (52, 53). Based on these studies, I believe the B-RAFV600E mutation may promote dedifferentiation in thyroid cancer cells and further drive tumor metastasis and worsen disease outcomes. Understanding this could help us explore potential therapeutic targets and provide more precise treatment options for thyroid cancer patients. Previous studies have identified various risk and protective factors for PTCDM. Future researchers can evaluate these factors’ correlations and evidence levels to develop a comprehensive PTCDM assessment tool, categorizing patients into low, medium, and high-risk groups (54). This predictive tool would help clinicians tailor screening and treatment plans for each group, enhancing early detection and targeted therapy while reducing unnecessary exams and treatments for low-risk patients, thus easing their financial and societal healthcare burdens.

In this study, 623 institutions published a total of 479 papers. Specifically, 491 of these institutions published only one paper on the topic. This indicates that high-output institutions are relatively rare in this research field. However, among the institutions that published eight or more papers, eight are located in China. This phenomenon is likely closely related to China’s large population base and the high incidence of PTC in the country. The incidence of PTC in China continues to rise, and even though the rate of distant metastases is relatively low, the sheer number of patients leads to a significant increase in the absolute number of PTCDM cases. This not only poses a significant burden on the public health system but also attracts considerable research interest and attention from scholars (55, 56).

There are several reasons why China and the United States dominate PTCDM research. First, funding is a crucial factor. The Chinese government and institutions like the National Institutes of Health (NIH) in the United States allocate substantial funds annually to cancer research, significantly boosting PTCDM research activities (57, 58). Additionally, the high incidence rates of cancer and thyroid diseases in these countries make PTCDM research a priority area (55). Both countries have numerous clinical hospitals and medical institutions, offering abundant case data and sample resources for research (57). This wealth of clinical data allows researchers to conduct large-scale clinical studies and experiments, enhancing the scientific rigor and reliability of their research. Furthermore, these nations possess advanced research infrastructure and technology, including high-throughput sequencing and PET imaging, providing powerful tools for exploring PTCDM mechanisms and treatments (59, 60). Moreover, the major research institutions in China and the United States have extensive international collaboration networks, which not only enhance research quality but also facilitate the exchange of knowledge and technology (61). In summary, the leading position of China and the United States in PTCDM research can be attributed to ample funding, government policy support, high incidence rates of PTC, rich clinical data, advanced research infrastructure, and extensive international collaboration. These factors collectively drive research progress in this field.

Through an analysis of the most commonly used keywords in the PTCDM research field, it is evident that the main topics include “cancer,” “papillary thyroid carcinoma,” “metastasis,” “expression,” and “survival.” Among these, “cancer” is the most frequently used, appearing 185 times, followed by “papillary thyroid carcinoma” with 156 uses. “Papillary thyroid carcinoma” serves as a core keyword, forming a cluster that includes “biomarkers,” “diagnosis,” and “mutation.” This cluster connects several other large clusters centered around “metastasis,” “cancer,” “follicular variant,” and “carcinoma.” The broader terms like “cancer” and “tumors” were primarily used in the early stages of research in this field, exploding in usage starting in 2004 and generally falling out of common use after 2015. They have been replaced by terms describing the progression of malignant tumors like “neoplasm metastasis,” “migration,” “invasion,” and “progression,” as well as terms related to the mechanisms of PTCDM development such as “biomarker,” “akt pathway,” and “braf v600e.” It’s not hard to surmise that early researchers primarily focused on PTC as a disease itself, while distant metastasis was considered just one manifestation and outcome of PTC. In recent years, researchers have increasingly focused on PTCDM, beginning to explore the mechanisms and causes of distant metastasis in hopes of finding effective treatments for these patients. Notably, “diffuse sclerosing variant” has become a frequently used keyword after 2020, possibly due to researchers finding that this subtype is more prone to distant metastasis (62).

In the field of PTCDM, top academic journals such as “Thyroid” and “Journal of Clinical Endocrinology & Metabolism” have played a crucial role in promoting high-quality basic and clinical research. The studies published in these journals have not only deepened the scientific community’s understanding of the disease mechanisms but have also spurred innovations and improvements in treatment methods, significantly enhancing patient quality of life (63–67). As the medical field increasingly emphasizes interdisciplinary research, the intersection of endocrinology, surgery, oncology, and molecular biology has facilitated comprehensive and profound discussions on the complexities of thyroid cancer. For example, advances in molecular biology have identified biomarkers for PTCDM, providing a theoretical basis for developing targeted treatment strategies (67–69). Open Access (OA) journals, such as “Oncology Letters” and “Frontiers in Endocrinology,” which are among the top 15 most cited journals, indicate a broader dissemination and faster global sharing of research findings. This trend significantly promotes international collaboration and the application of research results (70). The rise of precision medicine, particularly in designing personalized treatment plans, is expected to rely on the analysis and interpretation of big data, covering everything from genomics to clinical responses (5, 19, 71, 72).

In this study, dual-map overlay analysis of journals related to PTCDM literature was conducted to reveal patterns of knowledge flow and interconnections between disciplines. The results show that the citing journals are primarily concentrated in molecular biology, immunology, and clinical medicine. Research in these areas directly influences the development of both basic research and clinical treatment strategies for thyroid cancer. Conversely, the distribution of cited journals is mainly in molecular biology, genetics, and a broader range of health sciences, including health, nursing, and medicine. This indicates that the impact of thyroid cancer research has extended to a wider medical and health domain. Notably, journals in the categories of systems science, computing, and computer science are also frequently cited, reflecting the importance of computational methods and data analysis techniques in modern medical research, particularly the trends of machine learning and artificial intelligence in cancer genomics and personalized medicine (71, 73). The progress and development of medicine rely not only on doctors and nurses but also on the efforts of various sectors of society and interdisciplinary cooperation. For example, government officials who create regulations and collect clinical information do so to provide reference data for national decision-making. However, this extensive and diverse data can also be used for medical analysis and to identify findings that aid clinical decisions (74). In the PTCDM field, collaboration between molecular biologists and clinicians is particularly close, significantly advancing research in this area and providing more options for clinical decisions (34, 35, 75). This interdisciplinary flow of knowledge not only promotes the development of the thyroid cancer research field but also accelerates the application of new technologies and methods in medical research. With the application of artificial intelligence in data analysis and the development of advanced imaging and diagnostic technologies, researchers will be able to more accurately identify disease markers and optimize treatment strategies (76–78). It is expected that in the future, the PTCDM field will also closely collaborate with artificial intelligence. These advancements are expected to radically transform the landscape of thyroid cancer diagnosis and treatment in the future.

With scientific and technological advancements, interdisciplinary collaboration has become increasingly common. Molecular biology provides high-throughput sequencing data, while computer science and statistics analyze these data, advancing bioinformatics. The creation of R packages like limma, Seurat, and Monocle has equipped medical researchers with more analytical tools (79–81). Bioinformatics is essential for studying disease mechanisms, particularly in the PTCDM field.

In 2020, Lan X and colleagues identified chromosome 22q loss and gene fusions as biomarkers for predicting PTCDM through whole-exome sequencing (19). Similarly, R. Liu and others found that BRAF mutations in patients often co-occur with TERT promoter mutations, which are linked to aggressive features and higher recurrence or metastasis risk (82). Further research by Fudan University revealed that TERT promoter mutations accelerate BRAF mutations by regulating ribosome biogenesis, promoting PTC progression (83).Recent research has focused on integrating large language models (LLMs) with medicine. In March 2024, Shao-Hong Wu published a paper demonstrating the use of LLMs to enhance the consistency and accuracy of thyroid nodule malignancy diagnoses (84). This groundbreaking application suggests that future thyroid research will increasingly utilize LLM technology.

These achievements would not have been possible without the development of molecular biology and computer science. We can expect future medical research to increasingly leverage computer science and artificial intelligence to address scientific and clinical problems. However, new technologies also bring challenges, such as the ethical issues arising from LLMs and the ethical and security concerns associated with high-throughput sequencing. It is essential to develop and implement relevant regulations promptly to mitigate the potential consequences of these issues.

## Limitation

One limitation of this study is its reliance on bibliometric data from WoS databases, which might not encompass all relevant publications and could omit influential works not indexed within these systems. Additionally, the scope of this study is confined to English-language studies, and due to the ongoing updates to the database and exclusion of non-research articles, the results may not fully reflect the actual landscape. For more comprehensive outcomes, future research could extend searches to additional databases such as PubMed, Medline, Scopus, or Google Scholar.

In addition, although VOSviewer and CiteSpace are suitable tools for bibliometric analysis, they can introduce biases due to their reliance on keyword frequency and citation patterns. This may lead to the overrepresentation of certain keywords or research fields, particularly those frequently mentioned or cited, potentially skewing the analysis results (85).

To mitigate these limitations, we implemented several measures. We rigorously screened input data to minimize noise and irrelevant information. We used multiple clustering algorithms and parameters for cross-validation to ensure robustness. Additionally, during dual graph overlay analysis, we validated consistency and robustness by running the analysis multiple times with adjusted parameters. Despite these efforts, some sample source bias may remain as our data mainly comes from specific databases and journals. Nevertheless, we believe these technological and methodological improvements effectively reveal research dynamics and trends in the field of PTCDM.

## Conclusion

This study conducted a bibliometric analysis of 479 PTCDM-related publications from 2004 to 2023, revealing research trends and knowledge structures within the field. The analysis indicated that China, the USA, and South Korea are the most active countries in this research area, with Italy notable for its high citation rates. This leading position is attributed to substantial funding, high incidence rates, advanced research infrastructure, and extensive international collaboration. Key institutions such as China Medical University and Karolinska Institute have been identified as major contributors, providing significant insights and advancements in PTCDM research. Top journals like “Thyroid” and “Journal of Clinical Endocrinology & Metabolism” have played a pivotal role in promoting high-quality basic and clinical research, significantly fostering innovations and improvements in thyroid cancer treatment strategies. In the development of the research field, “BRAF,” “E-cadherin,” and the “AKT pathway” have emerged as three key research topics worth noting in recent years.

Furthermore, the study highlights the increasing importance of interdisciplinary approaches in the PTCDM field, particularly the integration of systems science, computer science, and artificial intelligence. These integrations provide new perspectives and technological paths for the diagnosis and treatment of thyroid cancer. These advancements not only accelerate innovation in treatment methods but also offer more precise treatment options for patients, potentially heralding a fundamental transformation in the management of this disease in the future. This study anticipates that future PTCDM research will increasingly integrate computer science and artificial intelligence, leading to more research outcomes. Researchers should consider applying these technologies in their PTCDM studies.

## Data Availability

The original contributions presented in the study are included in the article/supplementary material. Further inquiries can be directed to the corresponding authors.
